# Optimization of ATP synthase function in mitochondria and chloroplasts via the adenylate kinase equilibrium

**DOI:** 10.3389/fpls.2015.00010

**Published:** 2015-01-28

**Authors:** Abir U. Igamberdiev, Leszek A. Kleczkowski

**Affiliations:** ^1^Department of Biology, Memorial University of Newfoundland, St. John’s, NL, Canada; ^2^Department of Plant Physiology, Umeå Plant Science Centre, University of Umeå, Umeå, Sweden

**Keywords:** adenylate kinase, ATP synthase, chemiosmosis, chloroplasts, magnesium, mitochondria

## Abstract

The bulk of ATP synthesis in plants is performed by ATP synthase, the main bioenergetics engine of cells, operating both in mitochondria and in chloroplasts. The reaction mechanism of ATP synthase has been studied in detail for over half a century; however, its optimal performance depends also on the steady delivery of ATP synthase substrates and the removal of its products. For mitochondrial ATP synthase, we analyze here the provision of stable conditions for (i) the supply of ADP and Mg^2+^, supported by adenylate kinase (AK) equilibrium in the intermembrane space, (ii) the supply of phosphate via membrane transporter in symport with H^+^, and (iii) the conditions of outflow of ATP by adenylate transporter carrying out the exchange of free adenylates. We also show that, in chloroplasts, AK equilibrates adenylates and governs Mg^2+^ contents in the stroma, optimizing ATP synthase and Calvin cycle operation, and affecting the import of inorganic phosphate in exchange with triose phosphates. It is argued that chemiosmosis is not the sole component of ATP synthase performance, which also depends on AK-mediated equilibrium of adenylates and Mg^2+^, adenylate transport, and phosphate release and supply.

## INTRODUCTION

ATP synthase is the central bioenergetic engine of all organisms and represents the smallest molecular motor, which was optimized in the course of evolution. The knowledge about operation of ATP synthase has advanced significantly, and the coupling of electrochemical gradient via mechanical movement of catalytic subunits to the enzymatic catalysis of ATP production was studied in detail (Boyer, 1997). However, for optimal operation of this engine enzyme, it is important to consider its dynamic environment to keep optimal load of substrates and removal of products to maintain the stable non-equilibrium process of ATP synthesis. The reaction of ATP synthase requires delivery of protons, magnesium, ADP and phosphate, and consumption of formed ATP.

In eukaryotes, the ATP synthase complex is located in the inner membrane of mitochondria, with ATP synthesis reaction occurring on the membrane side toward matrix compartment. In plants, the enzyme is in addition localized in the thylakoid membrane of chloroplasts, with the ATP-forming-moiety facing the stroma. These topological differences between the mitochondrial and chloroplastic ATP synthases bring about two very distinct metabolic environments for ATP synthesis, where ATP utilization and provision of both ADP and Pi need to be fine-tuned for optimal ATP synthase activity. In chloroplasts, ATP synthase receives protons from thylakoid lumen, which volume is small as compared to the mitochondrial intermembrane space (IMS) and which pH value can drop to the values below 5 (Oja et al., 1999), while in the mitochondrial IMS it drops only slightly below 7 (Moore and Rich, 1985; Porcelli et al., 2005). In mitochondria, adenylates are transported through the membrane, whereas their stromal pool is self-sufficient to support chloroplastic ATP synthase; the activity of adenylate transport between chloroplast and cytosol is very low, representing ∼1% of activity of the triose phosphate translocator (Weber and Fischer, 2007).

While generation of proton electrochemical potential became the central theory in the chemiosmotic concept of ATP synthase operation (Mitchell, 1961), the optimal conditions of delivery of ADP and phosphate were analyzed in the concept of thermodynamic buffering (Stucki, 1980a,b), underlying the importance of auxiliary buffering enzymes such as adenylate kinase (AK) and creatine kinase in provision of the stable flux of ADP to ATP synthase. This theory was extended in relation to operation of AK in the IMS of mitochondria (Igamberdiev and Kleczkowski, 2009). In the present paper, we discuss how the equilibration of adenylates provides an optimal dynamic environment for operation of ATP synthase in mitochondria and chloroplasts, and for its optimized performance in living cell. The energy balance of photosynthetic cells is provided by equilibration of adenylate levels by chloroplasts and mitochondria (and the cytosol) and the role of AK in this equilibration appears to be important.

## BUFFERING OF PROTONS BY OTHER CATIONS IN ATP SYNTHESIS

According to the views of Mitchell (1961) and Williams (1961, 2011), the non-equilibrium hydrogen ion (proton) potentials are set up in biological phases by the electron transport chain (ETC). The membranes with associated proteins form a dynamic structure that is connected in activity by smaller organic molecules and metal ions, both of which may become virtually permanently bound, but many are in part free and mobile. When we consider proton gradient in mitochondria, there is not a very high difference in [H^+^] between the matrix and the IMS of mitochondria. This difference may be less than one pH unit and in plant mitochondria it was calculated as ∼0.3 in state 3 and ∼0.5 in state 4 (the matrix pH was determined as 7.3, and the IMS pH as 7.0 and 6.8 in state 3 and 4, respectively; Moore and Rich, 1985). Later studies performed on fully operational animal mitochondria estimate the matrix pH value at 7.6–8.1 and the IMS pH at 6.9, which corresponds to a pH gradient of 0.7–1.2 units (Porcelli et al., 2005; Wiederkehr, 2009). In the thylakoid lumen, which does not contain AK keeping control over free cation concentrations, the pH value can drop to the values of 5 and even lower (Oja et al., 1999). In these particular conditions, the transfer of protons to ATP synthase may not be vectorized and better corresponds to the classical Mitchell’s mechanism.

The value of pH 7 corresponds to the proton concentration of 0.1 μM, while the concentration of Mg^2+^ ions in the IMS is close to millimolar (with K^+^ even more abundant at ca. 100 mM; Williams, 2011). The concentration of Mg^2+^ under AK equilibrium at pH 7 in the IMS is close to 0.4 mM, i.e., 4000 times higher than the concentration of protons. This difference makes contribution of protons to the electrochemical gradient minimal, and release of Mg^2+^ in the AK reaction may serve as a tool preventing active dissipation of protons from ETC to the IMS, thus directing them to molecular targets, the most important being ATP synthase. In other words, the high concentration of K^+^, Mg^2+^, and other cations in the IMS represents a buffering mechanism preventing its drastic acidification [by transformation of ΔpH to membrane potential (Δψ)]. It is worth noting that Mg^2+^ release under AK equilibrium is facilitated at lower pH (Igamberdiev and Kleczkowski, 2003). Thus magnesium and other metals are important for buffering proton circuits. Magnesium (together with K^+^ and other abundant cations) buffers charge separation. However, the question remains whether the high concentration of K^+^ and the active K^+^/H^+^ antiport would not diminish the significance of Mg^2+^ in transforming ΔpH to Δψ. Other roles of magnesium will be discussed below.

The role of protons is also essential in prevention of futile ATP hydrolysis (Gaballo et al., 2002). In catalysis of ATP synthesis, the futile ATP hydrolysis by the F_1_F_o_ complex is inhibited by the ATPase inhibitor protein (IF_1_), which reversibly binds at one side of the F_1_F_o_ connection. The trans-membrane ΔpH component of the respiratory proton-motive force (PMF) displaces IF_1_ from the complex; in particular the matrix pH is the critical factor for IF_1_ association and its related inhibitory activity. Based on isotope-exchange experiments, enzyme-bound ATP is formed from ADP and Pi in the absence of a PMF (Boyer, 2000). It is the release of ATP (unbinding) that depends on the PMF. This is the most evident role of protons (driving ATP synthesis), and they also drive Pi influx (in symport) and (by decreasing pH) stimulate release of Mg^2+^ in the IMS in the AK reaction (Igamberdiev and Kleczkowski, 2003). This, by the accumulation of positive charge, will further buffer the ETC-driven release of protons to the IMS thus directing them to ATP synthase. Interestingly, mechanical energy alone (without the PMF) may also drive mitochondrial ATP formation via the ATP synthase reaction (Itoh et al., 2004).

## THE ROLE OF MAGNESIUM IN THE MECHANISM OF ATP SYNTHESIS

**FIGURE 1:**
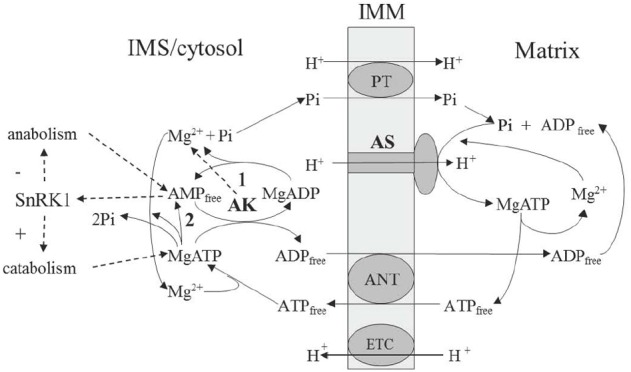
**Scheme of the AK involvement in supporting operation of the mitochondrial ATP synthase.** AMP-generating reactions include apyrase **(1)** and ATP-consuming biosynthetic reactions releasing AMP and pyrophosphate **(2)**. AK, adenylate kinase; ANT, adenine nucleotide translocator; AS, ATP synthase; ETC, electron transport chain; IMM, inner mitochochondrial membrane; PT, phosphate translocator; SnRK1, plant analog of AMP-activated protein kinase (sucrose-non-fermenting-1-related protein kinase-1).

The role of magnesium in ATP synthesis is underlined not only by the fact that MgATP is the actual product of the reaction, but also, as we show below, that Mg^2+^ acts as a separate substrate in the ATP synthase reaction (Figure 1). Under physiological conditions, ADP can exist both in a free and Mg-bound state, and this dual chemical capacity determines a way that magnesium becomes a part of “energy charge.” The Mg^2+^ pool is not less important than protons and it is generated (kept stable) by the AK reaction, which determines the equilibrium value of Mg^2+^ in cellular compartments (Igamberdiev and Kleczkowski, 2001). This results in efficient regulation of Mg-dependent enzymes and, as we show further, one such enzyme is ATP synthase.

The rotation mechanism of ATP synthase was suggested by Boyer (1989) and then it was demonstrated empirically (Noji et al., 1997). The role of proton translocation consists in deforming an open catalytic site to increase the affinity for ADP and Pi, which then bind and pass through the transition state, yielding tightly bound ATP in one binding change. ADP binding appears to be a key parameter controlling rotation during synthesis, while MgADP is inhibiting. The essential role of Mg^2+^ in ATP synthase catalysis was recently established in works of the laboratory of Pedersen. Previously it was assumed that the substrate of ATP synthase was MgADP (Boyer, 1997). Later studies, however, have indicated that it is free ADP in the presence of magnesium which represents the real substrate. It was shown (Ko et al., 1999) that inhibition of catalysis by vanadate in the presence of MgADP could be substituted by the Mg-vanadate complex indicating that Mg^2+^ plays a pivotal role in transition state formation during ATP synthesis. This state involves the preferential coordination with Pi and the repositioning of the P-loop to bring the non-polar alanine 158 into the catalytic pocket, which is achieved in the presence of Mg^2+^ (Blum et al., 2012). According to these more recent data, it is correct to consider ADP free rather than MgADP as a true substrate, while Mg^2+^ acts independently. Therefore, the substrates of ATP synthase are ADP free, Pi free, Mg^2+^ free, and H^+^, while the product is MgATP (Figure 1). The reaction can be presented by the following equation (one proton is the substrate, whereas other protons have catalytic function):

$${\text{ADP}}^{\text{3}}+{\text{HPO}}_{\text{4}}+{\text{Mg}}^{\text{2+}}+{\text{H}}^{\text{+}}\to {\text{MgATP}}^{\text{2}-}+{\text{H}}_{\text{2}}\text{O}$$

The difference in pH between matrix and IMS results in deprotonation of phosphate and of ADP in the matrix side, facilitating the Mg-dependent mechanism. Magnesium participating in ATP synthase catalysis exhibits a profound catalytic effect as shown by Buchachenko et al. (2008). The activity with ^25^Mg, which has magnetic isotopic nucleus, is two to three times higher than with ^24^Mg or ^26^Mg isotopes, having spinless non-magnetic isotopic nuclei. This suggests that the ATP synthesis is a spin-dependent ion-radical process. It implies a reversible electron transfer from the terminal phosphate of ADP^3–^ to Mg^2+^, generating ion-radical pair with singlet and triplet spin states. The yields of ATP along the singlet and triplet channels are controlled by hyperfine coupling of unpaired electron in ^25^Mg^+^ ion with magnetic nucleus. The magnesium bivalent cation transforms the protein molecule mechanics into a chemical reaction (Buchachenko et al., 2008). Although this mechanism was suggested for the mitochondrial ATP synthase, potentially it can be generalized for all ATP synthases including the chloroplast and even for other Mg-dependent enzymes.

Mg^2+^ uptake by mitochondria and its efflux are mediated by a channel or transporter responding to changes in membrane potential, in particular in pH gradient (Jung and Brierley, 1994; Li et al., 2008). The concentration of Mg^2+^ in the mitochondrial matrix depends on Pi which interacts strongly with Mg^2+^ to decrease its concentration and, in the absence of external Mg^2+^, promotes respiration-dependent Mg^2+^ efflux and its decrease in the matrix to very low levels (Jung et al., 1997). The uptake of Pi by respiring mitochondria converts ΔpH to Δψ and provides additional Mg-binding sites permitting its large accumulations. This means that Pi, in addition to AK, buffers Mg^2+^ concentration and this buffering is important in the matrix of plant mitochondria, where AK is absent.

While Mg^2+^ is an important catalyst (and substrate) of ATP synthesis and many other processes, the changes of its content result in significant shifts in bioenergetic state of the cell. These Mg^2+^-dependent shifts strongly affect Ca^2+^ concentration in the IMS (Gilli et al., 1998; Malmendal et al., 1999). Ca^2+^ uptake by mitochondria is inhibited by Mg^2+^ via a mixed-type inhibition in the process of multistate catalytic binding and interconversion, in which phosphate is also involved as a regulator (Pradhan et al., 2011). A frequently observed increase in [Ca^2+^] under stress conditions is, therefore, mediated by fluctuations in Mg^2+^ and results in activation of Ca^2+^ dependent stress-induced enzymes. Thus the signaling and metabolic roles of Ca^2+^ are under control of magnesium, phosphate and adenylate energy charge that establishes equilibrium Mg^2+^ concentration.

## EQUILIBRATION OF ADENYLATES OPTIMIZES ATP SYNTHESIS IN MITOCHONDRIA

For ATP synthase, maintenance of storage energy (conformational relaxation) state is supported via stable dynamic environment (buffering). Catalytic cycles should be supported by the optimal load and optimal consumption and, in mitochondria, this is achieved by the stable (buffered) influx of substrates and stable (buffered) efflux of products to/from the matrix compartment. As mentioned earlier, the substrates of ATP synthase are ADP, Pi and Mg^2+^, whereas the product is MgATP. Proton can be also considered as a substrate and, in addition, it provides symport of Pi during translocation through the membrane (Figure 1). The binding of substrate releases energy for conformational relaxation (Blumenfeld, 1983), thus this release should be optimized by the rate of substrate supply (called load in the thermodynamic buffering concept of Stucki, 1980a,b). The process of ATP synthesis has a fluctuating load conductance and needs a maximal buffering of the ADP supply. It was experimentally shown that the oxidative phosphorylation obeys linear and symmetric relations between flows and forces (Lemasters and Billica, 1981). It can operate at optimal efficiency only if the conductance of the load, i.e., the ATP utilizing reactions in a living cell, is exactly matched by the output conductance of oxidative phosphorylation (Stucki, 1980a).

To satisfy this condition and maintain a stable far from equilibrium regime, the reversible ATP-utilizing reaction catalyzed by AK acts as a thermodynamic buffer (Stucki, 1980b). The AK which is compartmentalized in the IMS of mitochondria acts as a filter by which the adenylate concentration is adjusted to a correct value before being handed over by adenylate translocator (Roberts et al., 1997). It acts as a linear energy converter maintaining the linearity of oxidative phosphorylation within a physiological range (Igamberdiev and Kleczkowski, 2009). It may seem that thermodynamic buffer enzymes dissipate huge amounts of energy, in case of AK by consuming significant portion of ATP molecules without using them for chemical synthesis; however, this is a cost for providing high efficiency of operation of ATP synthase by keeping it in prolonged state of conformational relaxation where energy can be efficiently directed to the chemical work (ATP synthesis). Due to this, linear non-equilibrium thermodynamics becomes applicable, and the metabolic flux of ATP generation becomes derivable from the concentrations of nucleoside phosphates and metal ions established under such equilibrium. Thus thermodynamic buffering represents the basic regulatory principle for the maintenance of a stable far from equilibrium regime with the minimal production of entropy. Filling buffer reservoirs corresponds to the accumulation of free energy and the buffering of energy intermediates is its most efficient source (Shnoll, 1979). The futile equilibration of product with substrate may be considered as a price for the maintenance of the energy-efficient process.

In an effort to examine, under multiple metabolic conditions, contributions of mitochondrial proteins to cellular ATP levels, screening of an RNAi library targeting over 1000 nuclear-encoded genes corresponding to mitochondria-localized proteins revealed that AK was a key regulator of ATP levels (Lanning et al., 2014). One isoform of AK (AK4), which is enzymatically inactive, *in vitro* interacts with mitochondrial ADP/ATP translocator (Liu et al., 2009) and regulates its activity protecting cells under stress. According to Dahnke and Tsai (1994), *K*cat of AK is 650 s^–1^ which is one order of magnitude higher than that of ATP synthase and this is essential for efficient equilibration of substrate and product as in the case of other enzymes (Igamberdiev and Roussel, 2012; Bykova et al., 2014; Igamberdiev et al., 2014). The role of AK in operation of ATP synthase is multifunctional: it provides the constant load of substrate (ADP) by equilibrating adenylates and supports “state 3” of respiring mitochondria, it supplies Mg^2+^ as a second substrate of ATP synthase, and, depending on the export of protons to the IMS, it adjusts the value of electrochemical gradient (Igamberdiev and Kleczkowski, 2003). Concentrations of other ions (e.g., K^+^) are also adjusted via AK equilibrium (Blair, 1970) providing additional regulatory role for respiration. Concentration of AMP established in this equilibrium is the main factor shifting cytosolic metabolism toward either catabolic or anabolic processes via regulation of AMP-activated protein kinase, which in plants is called SnRK1 (sucrose-non-fermenting-1-related protein kinase-1; Figure 1).

## TRANSPORT OF PHOSPHATE AND ADENYLATES IN MITOCHONDRIA

An important prerequisite of stable operation of ATP synthase is its coordination with function of two translocators, the adenylate translocator and the phosphate translocator. These proteins operate electrogenically, and the adenylate translocator exchanges free adenylates, while the phosphate translocator exchanges free phosphate in the symport with proton (or in the antiport with OH^–^). The electrical currents measured with the reconstituted adenylate translocator demonstrate electrogenic translocation of adenylates and charge shift of reorienting carrier sites (Klingenberg, 2008). The mitochondrial phosphate transporter makes it possible for a very rapid transport of most of the Pi used in ATP synthesis (Ferreira and Pedersen, 1993). It operates via electrochemical gradient of protons; however, it is likely that the unidirectional phosphate transport is catalyzed by H_2_PO_4_^–^/OH^–^ antiport rather than by H_2_PO_4_^–^/H^+^ symport and non-competitively inhibited by other anions (Stappen and Krämer, 1994). Since the inner membrane of mitochondria possesses electrical potential difference depending on the rate of proton pumping by electron transport, the adenylate transporter and other charge-moving processes, this affects the transport of adenylates and their equilibration by AK (Igamberdiev and Kleczkowski, 2003, 2006). In the absence of a membrane potential, the equilibrium concentrations of total adenylates will correspond to equimolar concentrations of free adenylates inside and outside mitochondria. Under the generation of a membrane potential, the gradient between free adenylate species is established according to the Nernst equation, and ATP free/ADP free ratio becomes lower inside and higher outside mitochondria. Under the steady flux of adenylates, the ratio between ATP/ADP outside and ATP/ADP inside a given compartment reflects the value of the membrane potential and drives ATP synthesis. Quantitative estimations of respective ATP/ADP ratios in the presence of Δψ at AK equilibrium are given in our earlier paper (Igamberdiev and Kleczkowski, 2003).

The involvement of AK in respiration is likely supported by apyrase, an Mg-dependent enzyme, which is ubiquitously distributed in different tissues and exists in several subcellular compartments, including a cytosol and IMS-confined isozymes (Flores-Herrera et al., 1999; Zancani et al., 2001; Igamberdiev and Kleczkowski, 2006). Apyrase can use ADP as substrate to produce AMP and Pi, with the former then used (together with ATP) by AK to produce ADP (Figure 1). Operation of AK and apyrase, which we defined as AK/apyrase cycle (Igamberdiev and Kleczkowski, 2006), has its energetic cost for the optimal ATP-synthase performance. Other sources of AMP include reactions leading to the formation of CoA-derivatives, activation of amino acids for protein synthesis, or nucleotide pyrophosphatase (Igamberdiev and Kleczkowski, 2006). They all support fairly high AMP concentration in the cytosol (15–20% of total adenylates; Stitt et al., 1982).

A very low [Mg^2+^] in the cytosol facilitates high ADP free/MgADP ratio, whereas in the matrix there is a very low ATP free/MgATP ratio. Thus, the adenylate carrier activity will be likely limited by availability of free ATP (on matrix side) but not free ADP (IMS side). Cytosolic ADP has recently been postulated as the key factor controlling respiration, with [Mg^2+^] mediating not only free ADP level in cytosol, but also adenylate exchange across the inner mitochondrial membrane (Gout et al., 2014). Thus the bioenergetic function of mitochondria is controlled from the outside (cytosol), whereas chloroplast appears as a more autonomous system supporting its ATP-generating function via the ratio of adenylates in its stroma. A high permeability of the outer membrane of mitochondria to small ions and molecules, while keeping AK and other enzymes compartmentalized inside IMS, facilitates the exchange of adenylates and Mg^2+^ between the IMS and cytosol.

## DYNAMIC ENVIRONMENT OF ATP SYNTHASE IN CHLOROPLASTS

**FIGURE 2:**
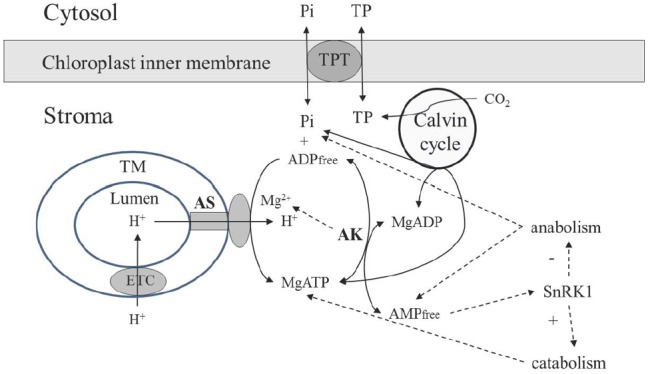
**Scheme of the AK involvement in supporting operation of the chloroplast ATP synthase.** Abbreviations are the same as in Figure 1. TM, thylakoid membrane; TPT, triose phosphate transporter.

The dynamic environment of ATP synthase in chloroplasts is established in a different (and in most aspects opposite) way as compared to mitochondria. ATP synthase receives protons from the thylakoid lumen (Figure 2), which has smaller volume as compared to the mitochondrial IMS, and its pH dropping to the values below 5 (Oja et al., 1999). The size of granal thylakoids was determined for *Arabidopsis* as 4 nm (stacking repeat distance) to 5 nm (diameter) in darkness, increasing to 19 nm in width and to 9–10 nm in diameter in the light (Kirchhoff et al., 2011), while the size of mitochondria is 500–1000 nm with the distance between two membranes 40–100 nm, depending on the physiological state and other factors (Sun et al., 2007). Adenylates are not supplied from lumen or IMS, the stromal pool is self-sufficient to support ATP synthase, and the activity of adenylate transport between chloroplast and cytosol is very low, representing ∼1% of activity of the triose phosphate translocator (Weber and Fischer, 2007). Although two chloroplast adenylate transporters were identified (Mohlmann et al., 1998), their role in photosynthetic tissues in chloroplasts is considered to be negligible, and they function to energize some processes in the stroma during the night rather than to transport ATP during photosynthesis (Weber and Fischer, 2007). Thus, it is quite certain that the stromal pool of adenylates is the sole source for AK-equilibrium governed delivery of ADP for ATP synthase reaction in chloroplasts.

There is no AK in thylakoid lumen, and the entire chloroplastic AK activity is confined to chloroplast stroma. Lange et al. (2008) has shown that the *Arabidopsis* genome contains 10 genes with an adenylate/cytidylate kinase signature; seven of them are identified as AK, two being targeted to plastids. Whereas silencing of the gene for one of the chloroplastic AK had no effect on plant phenotype, the second chloroplast AK was essential for proper growth and development. The absence of this protein caused only 30% reduction of total AK activity in leaves, but significantly affected chloroplast integrity and plant phenotype, resulting in small, pale-looking plantlets. Although to-date the importance of the first chloroplast AK isoform is not clear, the crucial role of the second is evident in providing proper chloroplast functioning and integrity. Several key metabolic processes are strongly affected by AK, e.g., poly (ADP-ribosyl)ation (Ishikawa et al., 2009).

Based on analyses of *K*_m_ values with adenylates for purified chloroplastic AK and on stromal contents of adenylates, the reaction of this enzyme is essentially displaced toward conversion of ATP and AMP to ADP (Schlattner et al., 1996). Thus, the AK reaction prevents over-accumulation of ATP, resulting in the balance of anabolic (Calvin cycle, starch synthesis, lipid biosynthesis, etc.) and catabolic reactions, in particular through dynamic maintenance of AMP concentration. The AMP-activated protein kinase (SnRK1) is highly active in chloroplasts (Fragoso et al., 2009), and the AMP concentration established under AK equilibrium plays a major role in shifting metabolism toward either biosynthetic or catabolic pathways (Figure 2). Both in rice and *Arabidopsis*, SnRK1 critically influences stress-inducible gene expression and the induction of stress tolerance, and its activity modulates plant developmental processes from early seedling development through late senescence (Cho et al., 2012).

Plants carrying out C_4_ metabolism (e.g., maize, sugarcane) have chloroplastic ATP synthase both in the mesophyll and bundle sheath cells (Majeran et al., 2008). This duality underscores different functions of chloroplastic ATP synthase in those cells. Whereas bundle sheath chloroplasts carry out the Calvin cycle and accumulate starch in the light, the mesophyll chloroplasts do not have Rubisco, and starch accumulation there ceases in mature leaves (Weise et al., 2011). Thus, in the mesophyll, the ATP formed by ATP-synthase must be linked to entirely different processes than in bundle sheath cells, and this occurs prominently by coupling to AK reaction. In C_4_ plants, the activity of AK from mesophyll cell chloroplasts is many-fold higher than in bundle sheath cells (Kleczkowski and Randall, 1986) and it is coupled to regeneration of phosphoenolpyruvate (PEP), the primary CO_2 _acceptor in C_4_ photosynthesis (Hatch, 1987). PEP is a product of pyruvate, Pi-dikinase reaction, which produces also AMP (and pyrophosphate). The AK uses this AMP as substrate, together with ATP produced by ATP synthase, and thus directly links the photophosphorylation rates in the mesophyll with primary CO_2_ fixation in those cells. A special function of AK and pyruvate, Pi-dikinase is evident also in C_3 _plants under anoxic conditions, where the joint operation of these enzymes provides an efficient use of PPi (in addition to ATP) as energy currency, thus avoiding drastic depletion of energy when mitochondrial respiration is suppressed by the lack of oxygen (Igamberdiev and Kleczkowski, 2011a,b).

**FIGURE 3:**
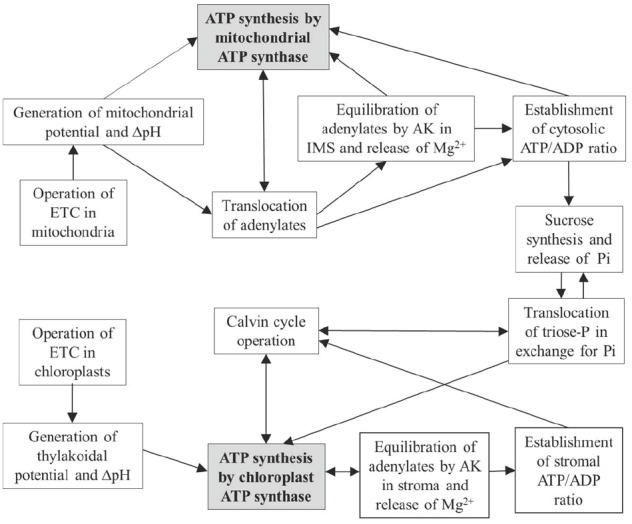
**Scheme linking operations of mitochondrial and chloroplastic ATP synthases with AK equilibrium in the IMS/cytosol and stroma compartments, and effects on subsequent carbon metabolism**.

Figure 3 shows how the interactions between chloroplasts and mitochondria (involving also cytosol) are optimized by operation of ATP synthase in the two compartments and by AK present in the chloroplast stroma and mitochondrial IMS. In chloroplasts, equilibration of adenylates in the stroma provides the establishment of ATP/ADP ratios for supporting the Calvin cycle, for maintenance of [Mg^2+^] and other cations, and for providing conditions for exchange of triose phosphates and inorganic phosphate between chloroplast and cytosol. In mitochondria, on the other hand, equilibration of adenylates takes place in the IMS, i.e., adenylate equilibrium is applied externally. This plays a role in supporting equilibrium concentrations of Mg^2+^ and other cations in the IMS, in optimization of adenylate transport between the mitochondrial matrix and cytosol, and also in establishment of the cytosolic ATP/ADP ratios governing operation of metabolic pathways in the cytosol, including sucrose synthesis, gluconeogenesis and other major metabolic processes. The depots of magnesium stored in vacuoles and mitochondria contribute via corresponding transporters (Shaul et al., 1999), in addition to the AK mechanism, to the establishment of equilibrium of Mg^2+^ in the cytosol.

## NON-COUPLED PATHWAYS

When it is not necessary for mitochondrial ATP synthase to further support ATP synthesis the non-coupled pathways of respiration become operative. The shifts in balance between the reactions of load and consumption that are beyond the buffering capacity of AK (and related mechanisms) can be adjusted via irreversible exergonic reactions that are not coupled to ATP synthesis. These reactions, at the first glance, can be described by the term “overflow” (Lambers, 1982); however they are tightly regulated and thus can be more correctly defined as the “regulated uncoupling” (Igamberdiev and Kleczkowski, 2009). They correspond to a slippage occurring when an enzyme passes a proton without ATP synthesis, e.g., the alternative oxidase (AOX) in the mitochondrial ETC in plants and fungi (Young et al., 2013). This slippage decreases the efficiency of energy utilization but enables controlling and regulating metabolic demands. Other non-coupled systems include the uncoupling proteins (UCPs; Vercesi et al., 2006) and rotenone-insensitive NAD(P)H dehydrogenases (Rasmusson et al., 2008). This type of thermodynamic buffering equilibrates the “energy charge” of the ATP pool with the redox charge of pyridine nucleotides. It prevents excessive proton pumping and thus buffers proton concentration for providing the optimal performance of the ATP synthase. It establishes the strict regulatory control of concentrations of NADH, NAD^+^ and ADP. The regulated uncoupling of NADH oxidation from ATP synthesis results in a buffered maintenance of the ATP/ADP and NADH/NAD^+^ ratios under different physiological conditions and thus keeping stable non-equilibrium state of ATP synthase (Igamberdiev and Kleczkowski, 2009).

In chloroplasts, there are many alternative sinks for electrons, and several of them are non-coupled with proton gradient (Ivanov et al., 2012), e.g., the role of chlororespiration, occurring via plastid terminal oxidase (PTOX; Houille-Vernes et al., 2011). The PTOX provides control of tuning of the redox state of electron carriers (Rumeau et al., 2007). However, these pathways are important mainly in preventing overreduction of chloroplast ETC and their capacity is insufficient for fine-tuning of redox and energy balance in the whole cell.

## CONCLUSION

We have presented evidence in this paper that the steady fluxes of adenylates, magnesium, hydrogen ions and phosphate established via thermodynamic buffering and regulated uncoupling support optimal load and consumption of ATP synthase and provide its stable catalytic cycle. AK equilibrium represents an essential bioenergetic regulatory principle for the maintenance of steady regimes of ATP synthesis in mitochondria and chloroplasts and its utilization in metabolic processes. This allows the maintenance of controlled rates of ATP production and consumption, in which the ratios of ATP and ADP, concentrations of Mg^2+^, the values of organellar membrane potential and hence the metabolic fluxes are tightly regulated. Despite of all similarities and differences in molecular regulation of ATP synthases in both mitochondria and chloroplasts, even though the topology is totally different, and despite the different location of AK in chloroplasts and mitochondria, in both cases the activities of ATP synthases are finely optimized. This optimization provides a dynamically stable homeostatic state essential for the maintenance of photosynthesis and for support of metabolic processes in plant cells and tissues.

### Conflict of Interest Statement

The authors declare that the research was conducted in the absence of any commercial or financial relationships that could be construed as a potential conflict of interest.

## References

[B1] Blair J. M. (1970). Magnesium, potassium, and the adenylate kinase equilibrium. Magnesium as a feedback signal from the adenine nucleotide pool. Eur. J. Biochem. 13, 384–390. 10.1111/j.1432-1033.1970.tb00940.x 4245368

[B2] Blum D. J. Ko Y. H. Pedersen P. L. (2012). Mitochondrial ATP synthase catalytic mechanism: a novel visual comparative structural approach emphasizes pivotal roles for Mg^2+^ and P-loop residues in making ATP. Biochemistry 51, 1532–1546. 10.1021/bi201595v 22243519

[B3] Blumenfeld L. A. (1983). Physics of Bioenergetic Processes. Berlin: Springer.

[B4] Boyer P. D. (1989). A perspective of the binding change mechanism for ATP synthesis. FASEB J. 3, 2164–2178. 2526771 10.1096/fasebj.3.10.2526771

[B5] Boyer P. D. (1997). The ATP synthase—a splendid molecular machine. Annu. Rev. Biochem. 66, 717–749. 10.1146/annurev.biochem.66.1.717 9242922

[B6] Boyer P. D. (2000). Catalytic site forms and controls in ATP synthase catalysis. Biochim. Biophys. Acta 1458, 252–262 10.1016/S0005-2728(00)00077-3 10838041

[B7] Buchachenko A. L. Kouznetsov D. A. Breslavskaya N. N. Orlova M. A. (2008). Magnesium isotope effects in enzymatic phosphorylation. J. Phys. Chem. B 112, 2548–2556. 10.1021/jp710989d 18247604

[B8] Bykova N. V. Møller I. M. Gardeström P. Igamberdiev A. U. (2014). The function of glycine decarboxylase complex is optimized to maintain high photorespiratory flux via buffering of its reaction products. Mitochondrion 19, 357–364. 10.1016/j.mito.2014.01.001 24444663

[B9] Cho Y. H. Hong J. W. Kim E. C. Yoo S. D. (2012). Regulatory functions of SnRK1 in stress-responsive gene expression and in plant growth and development. Plant Physiol. 158, 1955–1964. 10.1104/pp.111.189829 22232383 PMC3320198

[B10] Dahnke T. Tsai M. D. (1994). Mechanism of adenylate kinase. The conserved aspartates 140 and 141 are important for transition state stabilization instead of substrate-induced conformational changes. J. Biol. Chem. 269, 8075–8081. 8132532

[B11] Ferreira G. C. Pedersen P. L. (1993). Phosphate transport in mitochondria: past accomplishments, present problems, and future challenges. J. Bioenerg. Biomembr. 25, 483–492. 10.1007/BF01108405 8132488

[B12] Flores-Herrera O. Uribe A. Pardo J. P. Rendon J. L. Martinez F. (1999). A novel ATP-diphosphohydrolase from human term placental mitochondria. Placenta 20, 475–484. 10.1053/plac.1999.0401 10419813

[B13] Fragoso S. Espíndola L. Páez-Valencia J. Gamboa A. Camacho Y. Martínez-Barajas E. (2009). SnRK1 isoforms AKIN10 and AKIN11 are differentially regulated in *Arabidopsis* plants under phosphate starvation. Plant Physiol. 149, 1906–1916. 10.1104/pp.108.133298 19211700 PMC2663738

[B14] Gaballo A. Zanotti F. Papa S. (2002). Structures and interactions of proteins involved in the coupling function of the protonmotive FoF1-ATP synthase. Curr. Protein. Pept. Sci. 3, 451–460. 10.2174/1389203023380558 12370007

[B15] Gilli R. Lafitte D. Lopez C. Kilhoffer M. C. Makarov A. Briand C. (1998). Thermodynamic analysis of calcium and magnesium binding to calmodulin. Biochemistry 37, 5450–5456. 10.1021/bi972083a 9548926

[B16] Gout E. Rébeillé F. Douce R. Bligny R. (2014). Interplay of Mg^2+^, ADP, and ATP in the cytosol and mitochondria: unravelling the role of Mg^2+^ in cell respiration. Proc. Natl. Acad. Sci. U.S.A. 111, E4560–E4567. 10.1073/pnas.1406251111 25313036 PMC4217410

[B17] Hatch M. D. (1987). C4 photosynthesis—a unique blend of modified biochemistry, anatomy and ultrastructure. Biochim. Biophys. Acta 895, 81–106 10.1016/S0304-4173(87)80009-5

[B18] Houille-Vernes L. Rappaport F. Wollman F. A. Alric J. Johnson X. (2011). Plastid terminal oxidase 2 (PTOX2) is the major oxidase involved in chlororespiration in *Chlamydomonas*. Proc. Natl. Acad. Sci. U.S.A. 108, 20820–20825. 10.1073/pnas.1110518109 22143777 PMC3251066

[B19] Igamberdiev A. U. Kleczkowski L. A. (2001). Implications of adenylate kinase-governed equilibrium of adenylates on contents of free magnesium in plant cells and compartments. Biochem. J. 360, 225–231. 10.1042/0264-6021:3600225 11696011 PMC1222221

[B20] Igamberdiev A. U. Kleczkowski L. A. (2003). Membrane potential, adenylate levels and Mg^2+^ are interconnected via adenylate kinase equilibrium in plant cells. Biochim. Biophys. Acta 1607, 111–119. 10.1016/j.bbabio.2003.09.005 14670601

[B21] Igamberdiev A. U. Kleczkowski L. A. (2006). Equilibration of adenylates in the mitochondrial intermembrane space maintains respiration and regulates cytosolic metabolism. J. Exp. Bot. 57, 2133–2141. 10.1093/jxb/erl006 16798851

[B22] Igamberdiev A. U. Kleczkowski L. A. (2009). Metabolic systems maintain stable non-equilibrium via thermodynamic buffering. Bioessays 31, 1091–1099. 10.1002/bies.200900057 19708023

[B23] Igamberdiev A. U. Kleczkowski L. A. (2011a). Magnesium and cell energetics in plants under anoxia. Biochem. J. 437, 373–379. 10.1042/BJ20110213 21749322

[B24] Igamberdiev A. U. Kleczkowski L. A. (2011b). Optimization of CO_2_ fixation in photosynthetic cells via thermodynamic buffering. Biosystems 103, 224–229. 10.1016/j.biosystems.2010.10.001 20933572

[B25] Igamberdiev A. U. Roussel M. R. (2012). Feedforward non-Michaelis–Menten mechanism for CO_2_ uptake by Rubisco: contribution of carbonic anhydrases and photorespiration to optimization of photosynthetic carbon assimilation. Biosystems 107, 158–166. 10.1016/j.biosystems.2011.11.008 22154946

[B26] Igamberdiev A. U. Lernmark U. Gardeström P. (2014). Activity of the mitochondrial pyruvate dehydrogenase complex in plants is stimulated in the presence of malate. Mitochondrion 19, 184–190. 10.1016/j.mito.2014.04.006 24747677

[B27] Ishikawa K. Ogawa T. Hirosue E. Nakayama Y. Harada K. Fukusaki E. (2009). Modulation of the poly(ADP-ribosyl)ation reaction via the *Arabidopsis* ADP-ribose/NADH pyrophosphohydrolase, AtNUDX7, is involved in the response to oxidative stress. Plant Physiol. 151, 741–754 10.1104/pp.109.140442 19656905 PMC2754630

[B28] Itoh H. Takahashi A. Adachi K. Noji H. Yasuda R. Yoshida M. (2004). Mechanically driven ATP synthesis by F1 ATPase. Nature 427, 465–468. 10.1038/nature02212 14749837

[B29] Ivanov A. G. Rosso D. Savitch L. V. Stachula P. Rosembert M. Oquist G. (2012). Implications of alternative electron sinks in increased resistance of PSII and PSI photochemistry to high light stress in cold-acclimated *Arabidopsis thaliana*. Photosynth. Res. 113, 191–206. 10.1007/s11120-012-9769-y 22843101

[B30] Jung D. W. Brierley G. P. (1994). Magnesium transport by mitochondria. J. Bioenerg. Biomembr. 26, 527–535. 10.1007/BF00762737 7896768

[B31] Jung D. W. Panzeter E. Baysal K. Brierley G. P. (1997). On the relationship between matrix free Mg^2+^ concentration and total Mg^2+^ in heart mitochondria. Biochim. Biophys. Acta 1320, 310–320 10.1016/S0005-2728(97)00036-4 9230923

[B32] Kirchhoff H. Hall C. Wood M. Herbstová M. Tsabari O. Nevo R. (2011). Dynamic control of protein diffusion within the granal thylakoid lumen. Proc. Natl. Acad. Sci. U.S.A. 108, 20248–20253. 10.1073/pnas.1104141109 22128333 PMC3250138

[B33] Kleczkowski L. A. Randall D. D. (1986). Maize leaf adenylate kinase: purification and partial characterization. Plant Physiol. 81, 1110–1114. 10.1104/pp.81.4.1110 16664952 PMC1075494

[B34] Klingenberg M. (2008). The ADP and ATP transport in mitochondria and its carrier. Biochim. Biophys. Acta 1778, 1978–2021. 10.1016/j.bbamem.2008.04.011 18510943

[B35] Ko Y. H. Hong S. Pedersen P. L. (1999). Chemical mechanism of ATP synthase. Magnesium plays a pivotal role in formation of the transition state where ATP is synthesized from ADP and inorganic phosphate. J. Biol. Chem. 274, 28853–28856. 10.1074/jbc.274.41.28853 10506126

[B36] Lambers H. (1982). Cyanide-resistant respiration: a non-phosphorylating electron-transport pathway acting as an energy overflow. Physiol. Plant. 55, 478–485 10.1111/j.1399-3054.1982.tb04530.x

[B37] Lange P. R. Geserick C. Tischendorf G. Zrenner R. (2008). Functions of chloroplastic adenylate kinases in *Arabidopsis*. Plant Physiol. 146, 492–504. 10.1104/pp.107.114702 18162585 PMC2245825

[B38] Lanning N. J. Looyenga B. D. Kauffman A. L. Niemi N. M. Sudderth J. Deberardinis R. J. (2014). A mitochondrial RNAi screen defines cellular bioenergetic determinants and identifies an adenylate kinase as a key regulator of ATP levels. Cell Rep. 7, 907–917. 10.1016/j.celrep.2014.03.065 24767988 PMC4046887

[B39] Lemasters J. J. Billica W. H. (1981). Non-equilibrium thermodynamics of oxidative phosphorylation by inverted inner membrane vesicles of rat liver mitochondria. J. Biol. Chem. 256, 2949–2957. 7309743

[B40] Li L. G. Sokolov L. N. Yang Y. H. Li D. P. Ting J. Pandy G. K. (2008). A mitochondrial magnesium transporter functions in *Arabidopsis* pollen development. Mol. Plant 1, 675–685. 10.1093/mp/ssn031 19825572

[B41] Liu R. Ström A. L. Zhai J. Gal J. Bao S. Gong W. (2009). Enzymatically inactive adenylate kinase 4 interacts with mitochondrial ADP/ATP translocase. Int. J. Biochem. Cell Biol. 41, 1371–1380. 10.1016/j.biocel.2008.12.002 19130895 PMC2676352

[B42] Majeran W. Zybailov B. Ytterberg A. J. Dunsmore J. Sun Q. van Wijk K. J. (2008). Consequences of C4 differentiation for chloroplast membrane proteomes in maize mesophyll and bundle sheath cells. Mol. Cell. Proteomics 7, 1609–1638. 10.1074/mcp.M800016-MCP200 18453340 PMC2556027

[B43] Malmendal A. Linse S. Evenas J. Forsen S. Drakenberg T. (1999). Battle for EF-hands: magnesium-calcium interference in calmodulin. Biochemistry 38, 11844–11850. 10.1021/bi9909288 10512641

[B44] Mitchell P. (1961). Coupling of phosphorylation to electron and hydrogen transfer by a chemiosmotic mechanism. Nature 191, 144–146. 10.1038/191144a0 13771349

[B45] Mohlmann T. Tjaden J. Schwoppe C. Winkler H. H. Kampfenkel K. Neuhaus H. E. (1998). Occurrence of two plastidic ATP/ADP transporters in *Arabidopsis thaliana* L.—molecular characterisation and comparative structural analysis of similar ATP/ADP translocators from plastids and *Rickettsia prowazekii*. Eur. J. Biochem. 252, 353–359. 10.1046/j.1432-1327.1998.2520353.x 9546649

[B46] Moore A. L. Rich P. R. (1985). “Organization of the respiratory chain and oxidative phosphorylation,” in Encyclopedia of Plant Physiology, Higher Plant Cell Respiration, Vol. 18, eds Douce R. Day D. A. (Berlin: Springer), 134–172.

[B47] Noji H. Yasuda R. Yoshida M. Kinosita K. Jr. (1997). Direct observation of the rotation of F1-ATPase. Nature 386, 299–302. 10.1038/386299a0 9069291

[B48] Oja V. Savchenko G. Jakob B. Heber U. (1999). pH and buffer capacities of apoplastic and cytoplasmic cell compartments in leaves. Planta 209, 239–249. 10.1007/s004250050628 10436227

[B49] Porcelli A. M. Ghelli A. Zanna C. Pinton P. Rizzuto R. Rugolo M. (2005). pH difference across the outer mitochondrial membrane measured with a green fluorescent protein mutant. Biochem. Biophys. Res. Commun. 326, 799–804. 10.1016/j.bbrc.2004.11.105 15607740

[B50] Pradhan R. K. Qi F. Beard D. A. Dash R. K. (2011). Characterization of Mg^2+^ inhibition of mitochondrial Ca^2+^ uptake by a mechanistic model of mitochondrial Ca^2+^ uniporter. Biophys. J. 101, 2071–2081. 10.1016/j.bpj.2011.09.029 22067144 PMC3207172

[B51] Rasmusson A. G. Geisler D. A. Møller I. M. (2008). The multiplicity of dehydrogenases in the electron transport chain of plant mitochondria. Mitochondrion 8, 47–60. 10.1016/j.mito.2007.10.004 18033742

[B52] Roberts J. K. M. Aubert S. Gout E. Bligny R. Douce R. (1997). Cooperation and competition between adenylate kinase, nucleoside diphosphokinase, electron transport, and ATP synthase in plant mitochondria studied by 31P nuclear magnetic resonance. Plant Physiol. 113, 191–199. 12223600 10.1104/pp.113.1.191 PMC158130

[B53] Rumeau D. Peltier G. Cournac L. (2007). Chlororespiration and cyclic electron flow around PSI during photosynthesis and plant stress response. Plant Cell Environ. 30, 1041–1051. 10.1111/j.1365-3040.2007.01675.x 17661746

[B54] Schlattner U. Wagner E. Greppin H. Bonzon M. (1996). Chloroplast adenylate kinase from tobacco. Purification and partial characterization. Phytochemistry 42, 589–594 10.1016/0031-9422(95)00913-2

[B55] Shaul O. Hilgemann D. W. de-Almeida-Engler J. Van Montagu M. Inze D. Galili G. (1999). Cloning and characterization of a novel Mg^2+^/H^+^ exchanger. EMBO J. 18, 3973–3980. 10.1093/emboj/18.14.3973 10406802 PMC1171473

[B56] Shnoll S. E. (1979). Physico-Chemical Factors of Biological Evolution. Moscow: Nauka.

[B57] Stappen R. Krämer R. (1994). Kinetic mechanism of phosphate/phosphate and phosphate/OH^–^ antiports catalyzed by reconstituted phosphate carrier from beef heart mitochondria. J. Biol. Chem. 269, 11240–11246. 8157653

[B58] Stitt M. Lilley R. M. Heldt H. W. (1982). Adenine nucleotide levels in the cytosol, chloroplasts, and mitochondria of wheat leaf protoplasts. Plant Physiol. 70, 971–977. 10.1104/pp.70.4.971 16662653 PMC1065809

[B59] Stucki J. W. (1980a). The thermodynamic buffer enzymes. Eur. J. Biochem. 109, 257–267. 10.1111/j.1432-1033.1980.tb04791.x 7408880

[B60] Stucki J. W. (1980b). The optimal efficiency and the economic degrees of coupling of oxidative phosphorylation. Eur. J. Biochem. 109, 269–283. 10.1111/j.1432-1033.1980.tb04792.x 7408881

[B61] Sun M. G. Williams J. Munoz-Pinedo C. Perkins G. A. Brown J. M. Ellisman M. H. (2007). Correlated three-dimensional light and electron microscopy reveals transformation of mitochondria during apoptosis. Nature Cell Biol. 9, 1057–1065. 10.1038/ncb1630 17721514

[B62] Vercesi A. E. Borecký J. de Godoy Maia I. Arruda P. Cuccovia I. M. Chaimovich H. (2006). Plant uncoupling mitochondrial proteins. Annu. Rev. Plant Biol. 57, 383–404. 10.1146/annurev.arplant.57.032905.105335 16669767

[B63] Weber A. P. M. Fischer K. (2007). Making the connections—the crucial role of metabolite transporters at the interface between chloroplast and cytosol. FEBS Lett. 581, 2215–2222. 10.1016/j.febslet.2007.02.010 17316618

[B64] Weise S. E. van Wijk K. J. Sharkey T. D. (2011). The role of transitory starch in C3, CAM, and C4 metabolism and opportunities for engineering leaf starch accumulation. J. Exp. Bot. 62, 3109–3118. 10.1093/jxb/err035 21430293

[B65] Wiederkehr A. (2009). Matrix alkalinisation unleashes β-cell mitochondria. Islets 1, 154–156. 10.4161/isl.1.2.9058 21099264

[B66] Williams R. J. P. (1961). Possible functions of chains of catalysts. J. Theor. Biol. 1, 1–13 10.1016/0022-5193(61)90023-6 13785509

[B67] Williams R. J. P. (2011). Chemical advances in evolution by and changes in use of space during time. J. Theor. Biol. 268, 146–159. 10.1016/j.jtbi.2010.09.021 20869970

[B68] Young L. Shiba T. Harada S. Kita K. Albury M. S. Moore A. L. (2013). The alternative oxidases: simple oxidoreductase proteins with complex functions. Biochem. Soc. Trans. 41, 1305–1311. 10.1042/BST20130073 24059524

[B69] Zancani M. Casolo V. Vianello A. Macri F. (2001). Involvement of apyrase in the regulation of the adenylate pool by adenylate kinase in plant mitochondria. Plant Sci. 161, 927–933 10.1016/S0168-9452(01)00487-3

